# Study on HIF-1*α* Gene Translation in Psoriatic Epidermis with the Topical Treatment of Capsaicin Ointment

**DOI:** 10.5402/2011/821874

**Published:** 2011-06-22

**Authors:** Chun-shui Yu

**Affiliations:** Department of Dermatology, Affiliated Hospital of North Sichuan Medical College, Nanchong 637000, China

## Abstract

*Objective*. To investigate the mechanism of capsaicin in treating active psoriasis vulgaris. *Methods*. HIF-1*α* gene translation in active psoriatic lesions before and after 21-day treatment with capsaicin ointment was detected by *in situ* hybridization. *Results*. There was positive staining of HIF-1*α* gene in all the layers of psoriatic epidermis (100.0%) before the treatment with capsaicin ointment, but the dyeing in epidermis were reduced obviously (22.2%) after the treatment for 21 days. *Conclusion*. HIF-1*α* gene translation in psoriatic epidermis was downregulated after capsaicin treatment for 21 days.

## 1. Introduction

Psoriasis is a common skin disease characterized by hyperplastic regenerative epidermal growth and infiltration of immunocytes. The etiology of psoriasis is unknown although several genetic and cellular factors have been elucidated. Capsaicin is a naturally occurring substance derived from plants of the Solanaceae family (red peppers) with a kind of major pharmacologic effects on the peripheral part of the sensory nervous system, particularly on the primary afferent neurons of C-fiber type [1]. Hypoxia-inducible factor-1*α* (HIF-1*α*) is a more important factor in psoriatic epidermal proliferation. In order to find a new therapeutic method for psoriasis, we detected the translation of HIF-1*α* gene in psoriatic epidermis by *in situ* hybridization technique before and after 21 days treatment with capsaicin ointment. 

## 2. Materials and Methods

### 2.1. Subjects

A total of 42 patients (19 males and 23 females, aged from 18 to 52 years with the mean age of 34.2 years old) with active psoriasis vulgaris diagnosed by histology and clinical features were given either placebo or 0.025% capsaicin ointment three times daily for 21 days randomly by double-blind method. The treatment was agreed by all the patients. The therapeutic group consisted of 22 patients (10 males and 12 females, ranged from 19 to 52 years old with the mean of 31.7 years). The control group had 20 patients (9 males and 11 females, ranged from 18 to 40 years with the mean of 30.4 years). None of the patients had received topical or systemic treatment within the past 4 weeks. There was no significant difference between the two groups, and the sex and age for these samples of controls were exactly the same as those for the samples of the patients (*P* > .05). 

### 2.2. Methods

Each sample was immediately frozen in powdered dry ice after the operation and stored in a deep freezer at −80°C until use. The samples were fixed with 4% paraformaldehyde over night, paraffin embedded, and serially sectioned in 4 *μ*m thickness.

The samples were stained by HE to show their pathologic characters, then, were stained by *in situ* hybridization for observation of HIF-1*α* mRNA translation. The staining procedure was performed according to the operating instructions of the test kit.

 HIF-1*α* search was used to choose the appropriate sequences. The selected sequences showed no significant similarity with other sequences in the database. The following 5′-digoxigenin-labeled oligonucleotides were synthesized. HIF-1*α* probes: 5′-GGAAG TGGCA ACTGA TGAGC AAGCT CATAA-3′. For controls, the 5′-digoxigenin-labeled sequences of the corresponding mRNA were used.

With HIF-1*αin situ* hybridization detection kit (Sigma), the samples were routinely deparaffinized to deactivate endogenetic peroxide enzymes digested by protease K for 5 minutes. After the cDNA double chain was degenerated, the samples were placed into *in situ *hybridization solution of HIF-1*α* containing digoxin-labeled probes (Sigma) and hybridized for 12 to 16 hours at 37°C. The samples without probes were counted as the negative control group, also called the blank control. 

The violet blue granules products were displayed positively in cell cytoplasm, indicating the translation of HIF-1*α* mRNA. 

### 2.3. Statistical Analysis

Data were analyzed with the statistical software SPSS11.0. The statistical significance of differences between groups was tested by *χ* ^2^ test. *P* value less than  .05 was considered to be statistically significant. 

## 3. Results

### 3.1. Staining of In Situ Hybridization

**Figure 1:**
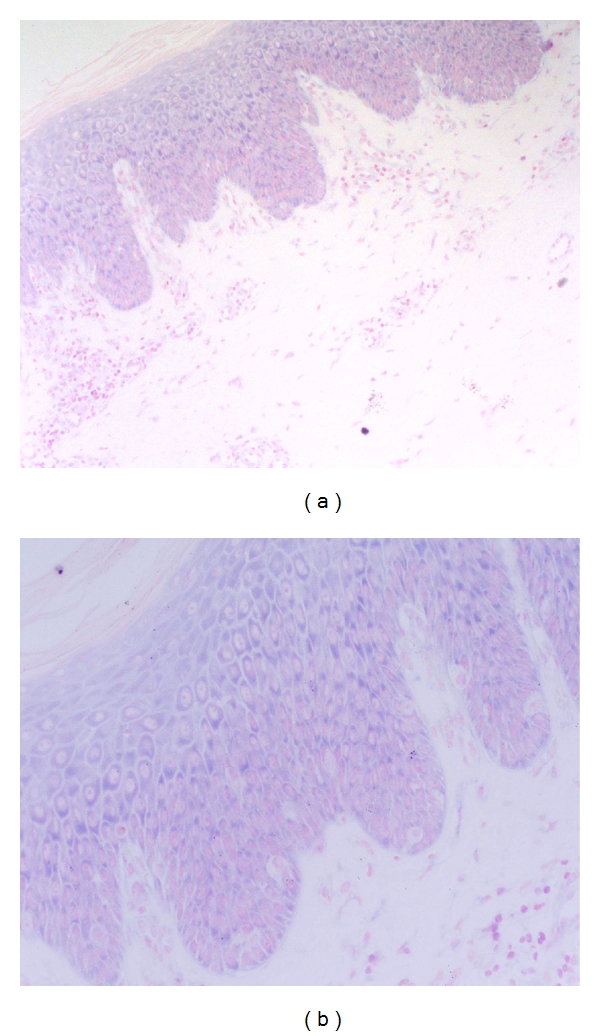
Before capsaicin treatment, there were positive violet blue granules staining in psoriatic epidermis (×200, ×400).

**Figure 2:**
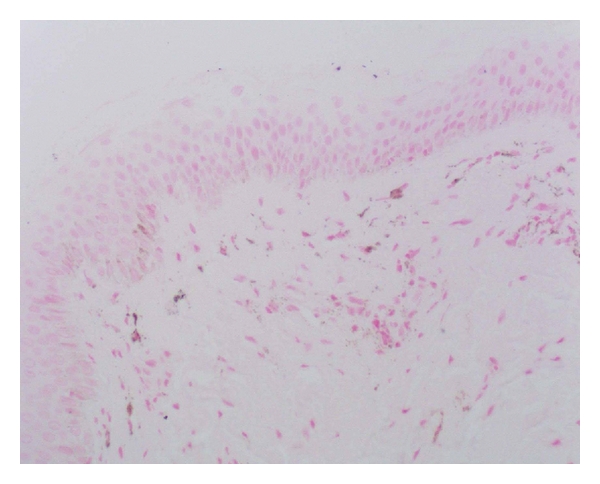
After topical treatment with capsaicin, there was nearly no violet blue granules staining of HIF-1*α* mRNA in psoriatic epidermis (×200).

Before the treatment with capsaicin ointment, the violet blue granules staining could be seen in all the layers of epidermis of psoriatic lesions in cytoplasm of positive cells (Figure 1). After 21-days topical treatment with capsaicin, there was no HIF-1*α* mRNA positive staining in all the layers of epidermis in most of the psoriatic lesions (18/22) (Figure 2). There were nearly no violet blue granules staining before and after treatment in control group. The translation of HIF-1*α* mRNA in epidermis of different groups is shown in Table 1. 

### 3.2. Translation of HIF-1*α* mRNA in the Tissues

Before capsaicin treatment, the positive rate of HIF-1*α* mRNA in all the layers of psoriatic epidermis was 22/22 (100.0%) and 1/20 (5.0%) in control group; after the treatment, the positive rate in psoriatic epidermis was 4/22 (18.2%) and 0/20 (0%) in control group. There was a significant difference between the two groups both before treatment (*χ* ^2^ = 38.17, *P* < .05) and after (*χ* ^2^ = 4.02, *P* < .05). 

## 4. Discussion

Psoriasis is a polygenetic hereditary multifactorial disease which may be influenced by a number of environmental factors. Today, only a few studies have experimentally investigated the influence of HIF-1*α* on psoriasis. So, in this study, we examined the effect and mechanism of HIF-1*α* on the proliferation of epidermis of psoriasis. Before and after 21-day treatment with capsaicin ointment, HIF-1*α* mRNA was detected by* in situ* hybridization.

Capsaicin is a naturally occurring substance derived from plants of the Solanaceae family (red peppers) and has the chemical name 8-methyl-N-vanillyl-6-nonenamide. It plays a role in preventing the development of thermal hyperalgesia in neonatal rats [1].

Transient receptor potential vanilloid receptor subtype 1 (TRPV1) is a member of TRP family; it is activated by capsaicin, heat, and protons and can be hypersensitized by injury. TRPV1 expression has been identified in nonneuronal cells as well as neuronal cells, for example, in bronchial epithelial cells, cardiomyocytes, urinary bladder epithelial cells, gastric epithelial cells, oral epithelium, and keratinocytes. The presence of functional TRPV1 receptors in keratinocytes has generated interest in skin-nerve crosstalk under physiological and pathological conditions. Activation of TRPV1 in human keratinocytes by capsaicin results in a dose-dependent expression of cyclooxygenase-2 [2]. It has been shown that neurogenic inflammation induced by capsaicin in patients with psoriasis [3]. TRPV1, a heat-gated channel, was recently found on human keratinocytes, and the activation of epidermal TRPV1 was known to induce release of proinflammatory mediators [4]. So, when applied topically to the skin of experimental humans, capsaicin maybe through TRPV1 receptor of keratinocytes, then, affect cells' function. 

Our studies clearly showed that, after the treatment with capsaicin, the translation of HIF-1*α* mRNA was clearly reduced in all the layers of psoriatic epidermis. From this, it can be seen that capsaicin may regulate the translation of HIF-1*α* mRNA through TRPV1 receptor, thereby inhibiting the hyperproliferation of psoriatic epidermis and inducing normal differentiation. 

After the treatment with capsaicin in psoriatic lesions, it downregulates the translation of HIF-1*α* mRNA, which induces the transcription of HIF-1*α* mRNA in all the layers of psoriatic epidermis and plays a role in inhibiting proliferation of keratinocytes. It has been shown that the expressions of HIF-1*α* protein were very weak in the control skin but very strong in psoriatic lesions. HIF-1*α* have high expression in psoriasis and might play an important role in the genesis and development of psoriasis [5].

Our results suggest that downregulation of HIF-1*α* mRNA by capsaicin may induce the proliferation and cytokine production of keratinocytes; this may be involved in the pathogenesis of psoriasis.

In conclusion, there was positive translation of HIF-1*α* mRNA in psoriatic epidermis, while the translation of HIF-1*α* mRNA was reduced after treatment with capsaicin. HIF-1*α* mRNA located in all layers of psoriatic epidermis could induce proliferation and differentiation of psoriatic epidermis keratinocytes, which made the epidermis thickened; with the recovery of the clinical symptoms, the translation of HIF-1*α* mRNA was decreased. Capsaicin inhibits proliferation and induces differentiation of keratinocytes through downregulating translation of HIF-1*α* mRNA in psoriatic epidermis. 

## Figures and Tables

**Table 1:** Translation of HIF-1*α* mRNA in different groups.

		Therapeutic group	Control group
Before treatment	Positive	22	1
	Negative	0	19*
After treatment	Positive	4	0
	Negative	18	20*

*P < .05, versus control.
